# MMP9 mediates acute hyperglycemia-induced human cardiac stem cell death by upregulating apoptosis and pyroptosis in vitro

**DOI:** 10.1038/s41419-020-2367-6

**Published:** 2020-03-13

**Authors:** Santosh K. Yadav, Tyler N. Kambis, Sumit Kar, Song Y. Park, Paras K. Mishra

**Affiliations:** 1 Department of Cellular and Integrative Physiology, University of Nebraska Medical Center, Omaha, NE 985850 USA; 2 Department of Health and Kinesiology, University of Nebraska at Omaha, Omaha, NE USA

**Keywords:** Stem cells, Heart stem cells

## Abstract

Providing a conducive microenvironment is critical to increase survival of transplanted stem cells in regenerative therapy. Hyperglycemia promotes stem cell death impairing cardiac regeneration in the diabetic heart. Understanding the molecular mechanisms of high glucose-induced stem cell death is important for improving cardiac regeneration in diabetic patients. Matrix metalloproteinase-9 (MMP9), a collagenase, is upregulated in the diabetic heart, and ablation of MMP9 decreases infarct size in the non-diabetic myocardial infarction heart. In the present study, we aim to investigate whether MMP9 is a mediator of hyperglycemia-induced cell death in human cardiac stem cells (hCSCs) in vitro. We created MMP9^−/−^ hCSCs to test the hypothesis that MMP9 mediates hyperglycemia-induced oxidative stress and cell death via apoptosis and pyroptosis in hCSCs, which is attenuated by the lack of MMP9. We found that hyperglycemia induced oxidative stress and increased cell death by promoting pyroptosis and apoptosis in hCSCs, which was prevented in MMP9^−/−^ hCSCs. These findings revealed a novel intracellular role of MMP9 in mediating stem cell death and provide a platform to assess whether MMP9 inhibition could improve hCSCs survival in stem cell therapy at least in acute hyperglycemic microenvironment.

## Introduction

Stem cells have potential to differentiate into multiple cell types, which is crucial for cardiac repair in regenerative therapy. However, a key challenge in stem cell therapy is death of transplanted stem cells in the damaged tissue microenvironment. This problem is amplified by diabetes mellitus (DM) where stem cell death is exacerbated by oxidative stress due to hyperglycemic microenvironment^1^. Thus, it is important to investigate the molecular mechanisms underlying hyperglycemia-induced cell death of human cardiac stem cells (hCSCs), which is thus far poorly understood. The most extensively studied form of cell death is apoptosis, where cytochrome c released from mitochondria forms apoptosome, which then activates caspase-3 leading to cell death^2^. DM damages mitochondria^3^ and damaged mitochondria release oxidized mitochondrial DNA that induces pyroptosis, a non-apoptotic, caspase-1-dependent, inflammatory cell death^2,4^. However, molecular mediators of apoptosis and pyroptosis are unclear in hCSCs in the hyperglycemic microenvironment.

Matrix metalloproteinases (MMPs) play crucial roles in signaling pathways of migration, proliferation, and differentiation of stem cells^5^. Matrix metalloproteinase-9 (MMP9) is involved in the process of colonization and migration of hematopoietic stem/progenitor cells into a secondary niche by regulating chemokines, cellular interactions, and signaling^6^. However, the intracellular function of MMP9 in stem cells is nebulous. The hyperglycemic microenvironment upregulates MMP9 in embryonic stem cells (ESCs). Expression of MMP9 is increased in undifferentiated ESCs but decreased in differentiated ESCs^7^. MMP9 ablation decreases infarct size after ischemia/reperfusion injury in the heart^8^. Because a decrease in myocardial infarct size indicates increased survival of myocardial tissue, it is assumed that MMP9 is potentially involved in cell death mechanisms, thus ablation of MMP9 improved survival of the myocardium. We also found robust upregulation of miR-133a in the MMP9^−/−^ heart^9^. MiR-133a promotes stem cell survival^10^ and inhibits apoptosis^11^. MMP9 is upregulated in the DM heart and absence of MMP9 in the DM heart is cardioprotective^9,12^. In addition, MMP9 modulates inflammation^13^ and promotes release of inflammatory cytokine interleukin-1β (IL-1β)^14^, which is involved in pyroptotic cell death^4^. However, it is unclear whether MMP9 is a mediator of hyperglycemia-induced stem cell death via apoptosis and pyroptosis.

In the present study, we sought to determine whether MMP9 mediates hyperglycemia-induced cell death via apoptosis and pyroptosis in hCSCs, and if attenuation of MMP9 improves viability of hCSCs.

## Results

### Characterization of hCSCs

**Fig. 1:**
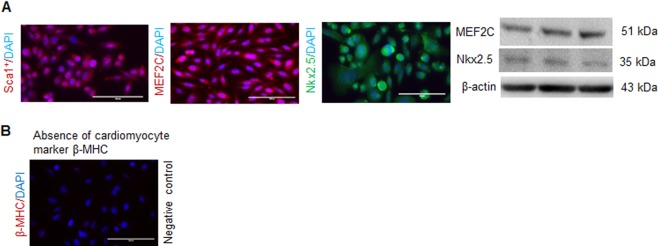
Validation of human cardiac stem cells (hCSCs). Experiments were performed to validate hCSCs using company reported markers. **a** Immunofluorescence staining for Sca1+, MEF2C, and NKX2.5. Western blotting for MEF2C and Nkx2.5. β-actin was used as a loading control. Lane 1, 2, and 3 are three different samples. **b** Immunofluorescence of cardiomyocyte marker β-MHC. It is a negative control showing absence of differentiated hCSCs.

In order to characterize commercially obtained hCSCs (human adult ventricular cardiac stem cells), we performed immunofluorescence and Western blotting, and confirmed the presence of key hCSC markers (Sca1^+^, MEF2C, Nkx2.5) (Fig. 1a), which were reported by the company (https://www.creative-bioarray.com/QualiCell®-Human-Adult-Ventricular-Cardiac-Stem-Cells-XLC452-CSC-C00305-item-2861.htm). To rule out the possibility of differentiated hCSCs, we evaluated cellular expression of β-myosin heavy chain (β-MHC), a marker of cardiomyocytes, which was absent in hCSCs (Fig. 1b). These findings support that the experimental cells were undifferentiated hCSCs.

### High glucose increases cell death and MMP9 activity in hCSCs

**Fig. 2:**
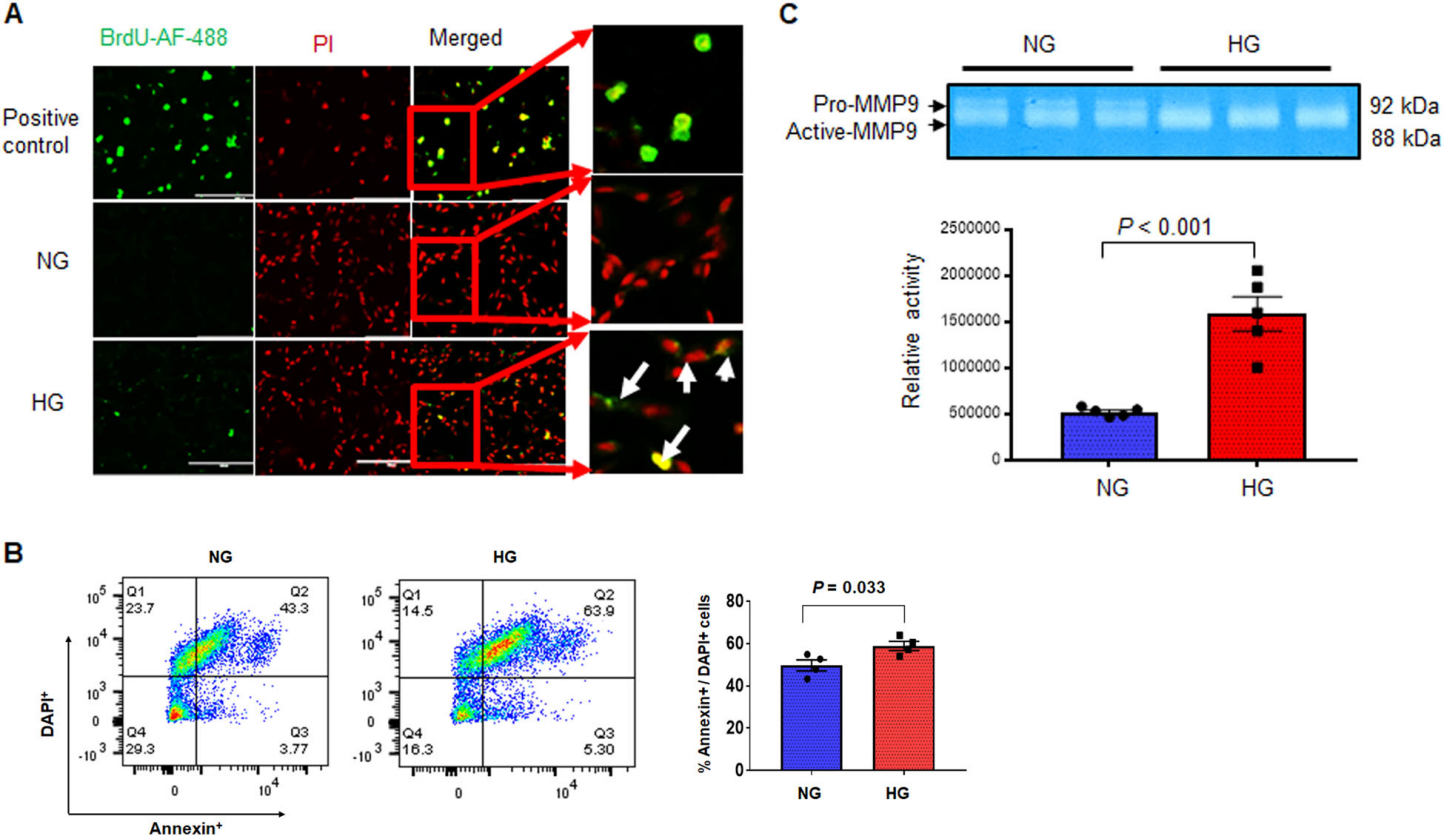
Hyperglycemic microenvironment increases cell death and MMP9 activity in hCSCs. The hCSCs were treated with normal (NG) or high (HG) dose of glucose for 24 h and subsequently used for analyses of cell death and MMP9 activity. **a** Representative TUNEL assay for apoptosis in hCSCs. TNFα + cyclohexamide treatment was a positive control for TUNEL. **b** Flow cytometry assay for Annexin V/DAPI, a marker of cell death, in HG or NG medium. **c** In-gel-gelatin zymography showing increased activity of MMP9 in hyperglycemic hCSCs. Values are expressed as mean ± SEM. Each dot represents one sample. Student *t*-test was used. *P* < 0.05 is considered statistically significant.

To determine whether hyperglycemic microenvironment increases hCSCs death, we incubated hCSCs with high (HG) or normal (NG) glucose medium for 24 h, and measured TUNEL positive cells, a marker of DNA damage and cell death. We used 10 µg/ml cyclohexamide + 30 ng/ml tumor necrosis factor-α (TNFα) as a positive control. We observed an increased TUNEL positive hCSCs in the HG group, demonstrating hyperglycemia-induced cell death in hCSCs (Fig. 2a). To corroborate this finding, we quantified Annexin V, a marker of cell death, by flow cytometry in HG and NG-treated hCSCs. The percentage of Annexin V was increased in the HG compared to the NG group (Fig. 2b), supporting that acute hyperglycemia promotes cell death in hCSCs.

To investigate whether increased hCSCs death is associated with MMP9 activation, we measured MMP9 activity in NG and HG groups by in-gel-gelatin zymography. We found increased MMP9 activity in the HG-treated hCSCs (Fig. 2c). These findings suggest that acute hyperglycemic microenvironment activates MMP9 concomitant with induction of cell death in hCSCs.

### Creation and validation of MMP9^−/−^ hCSCs

**Fig. 3:**
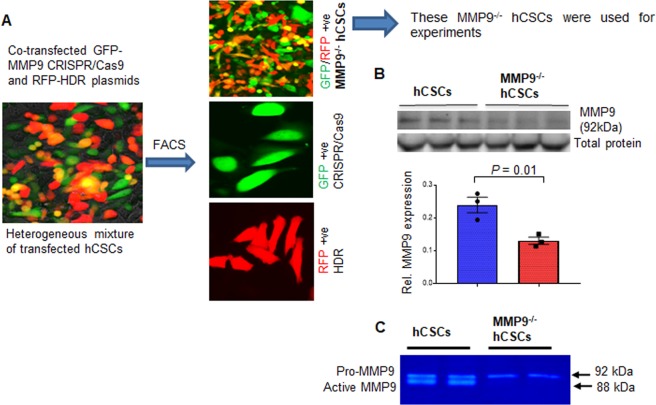
Creation and validation of MMP9^−/−^ hCSCs. CRISPR-Cas9 method was used to generate MMP9^−/−^ hCSCs. **a** Representative image showing heterogeneous hCSCs after co-transfection with CRISPR-Cas9 (green) and HDR (red) plasmids. Representative images of three groups of hCSCs after fluorescence-activated cell sorting (FACS). **b** Western blotting and densitometric qualification of MMP9 protein validating decreased MMP9 expression in MMP9 ablated hCSCs (MMP9^−/−^ hCSCs). β-actin is a loading control. Each dot represents one sample. Values are expressed as mean ± SEM. Student *t*-test was used. *P* < 0.05 is considered statistically significant. **c** Representative in-gel-gelatin zymography showing decreased MMP9 activity in MMP9^−/−^ hCSCs.

To establish the role of MMP9 in hyperglycemia-induced hCSCs death, we created MMP9^−/−^ hCSCs using the CRISPR-Cas9 technique (Fig. 3a). We co-transfected hCSCs with GFP (green)-tagged MMP9 CRISPR-Cas9 and RFP (red)-tagged homology-directed repair (HDR) plasmids. This generated heterogeneous population of differentially transfected hCSCs. We assorted GFP/RFP positive MMP9^−/−^ hCSCs by fluorescence-activated cell sorting (FACS), and used them for validation of MMP9^−/−^ (Fig. 3a). Of note, getting complete knockout by the CRISPR-Cas9 method in vitro is difficult. Thus, we do not rule out the possibility of obtaining heterozygous knockout cells. To determine whether MMP9 was knocked down in MMP9^−/−^ hCSCs, we measured the expression and activity of MMP9 in MMP9^−/−^ hCSCs and compared them to hCSCs. Our results showed decreased expression (Fig. 3b) and activity (Fig. 3c) of MMP9 in MMP9^−/−^ hCSCs.

### MMP9 mediates hyperglycemia-induced cell death and ablation of MMP9 improves viability of hCSCs

To determine whether MMP9 mediates hyperglycemia-induced hCSCs death and if abrogation of MMP9 improves viability of hCSCs, we treated hCSCs and MMP9^−/−^ hCSCs with NG or HG for 24 h. We evaluated key molecular markers involved in cell death signaling via apoptosis and pyroptosis, and determined cell viability in the treated cells.

#### A) Ablation of MMP9 blocks HG-induced apoptosis in hCSCs

**Fig. 4:**
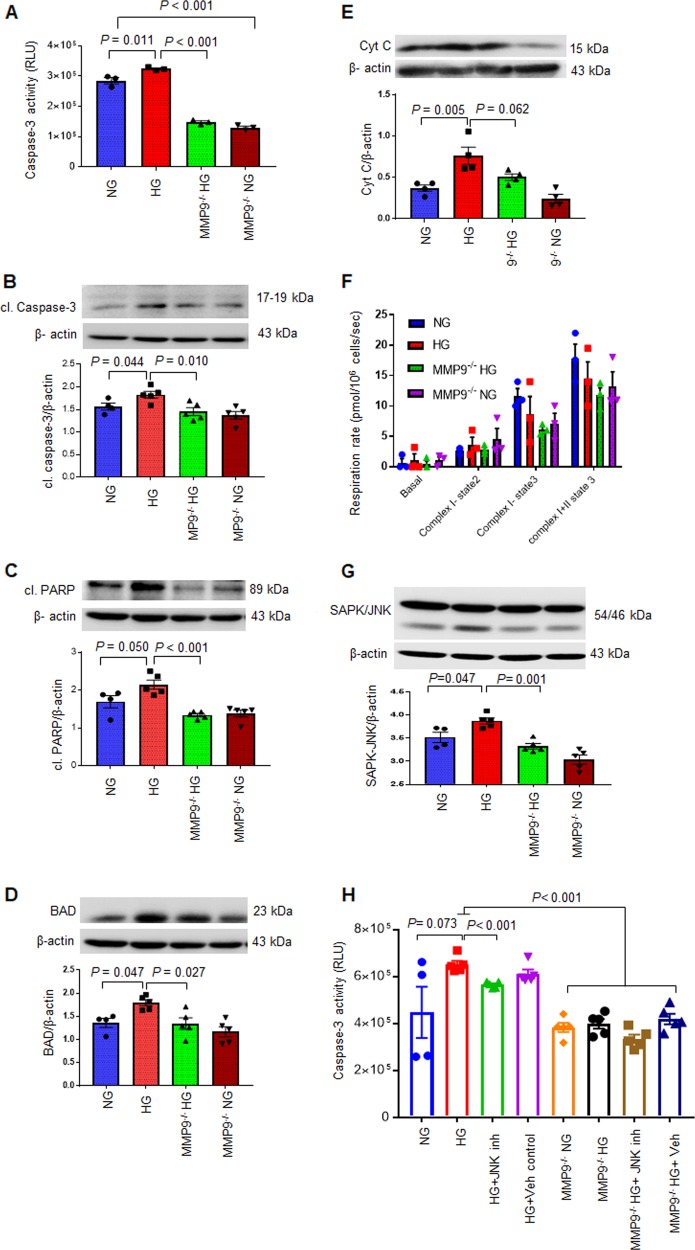
Absence of MMP9 blocks hyperglycemia-induced apoptosis without affecting mitochondrial function in hCSCs. Both hCSCs and MMP9^−/−^ hCSCs were incubated with normal (NG) or hyperglycemic (HG) culture medium for 24 h, and evaluated for apoptosis and mitochondrial function. **a** Caspase- Glo 3/7 assay kit was used to measure caspase3/7 activity. Relative luminescence unit (RLU) was used to evaluate differences in cascapse-3 activity. **b**–**e** Western blotting and densitometric analyses of apoptotic markers: cleaved (cl.) caspase-3, cl. PARP, BAD, and cytochrome c. **f** Evaluation of mitochondrial function by measuring respiration rate in different mitochondrial complexes. **g** Western blotting for SAPK-JNK. β-actin is a loading control. **h** Caspase-3/7 activity in HG or NG in the presence of JNK inhibitor/vehicle. Each dot represents one sample. Values are expressed as mean ± SEM. One-way ANOVA and Tukey’s post-hoc test was used. *P* < 0.05 is considered statistically significant.

We measured apoptotic cell death in the above-mentioned groups and found that HG increased caspase-3 activity (Fig. 4a) and expression (Fig. 4b)—a key molecular marker of apoptosis. HG also promoted other apoptosis molecular markers in hCSCs such as cleaved PARP (Fig. 4c), upregulated BAD (Fig. 4d), and increased levels of cytochrome c (Fig. 4e). All of these molecules were either downregulated or normalized in MMP9^−/−^ hCSCs (Fig. 4a–e), demonstrating that MMP9 mediates hyperglycemia-induced apoptosis in hCSCs. Notably, there was no difference in apoptosis markers between the HG and the NG group in MMP9^−/−^ hCSCs (Fig. 4a–d). Moreover, there was dramatic downregulation of caspase-3 activity in the NG group of MMP9^−/−^ hCSCs versus hCSCs (Fig. 4a), which elicits that MMP9 could be important for initiation of apoptosis irrespective of hyperglycemia. These findings elucidate the role of a hyperglycemic environment on apoptosis and establish MMP9 as a mediator of apoptosis in hCSCs.

Cytochrome c was slightly increased (statistically not significant) in HG as compared to NG group in MMP9^−/−^ hCSCs (Fig. 4e). This points to the possibility of a differential role of MMP9 in cytochrome c release from mitochondria in hyperglycemic microenvironment in hCSCs. Because increased cytochrome c release is associated with damaged mitochondria^15^, we determined whether MMP9 ablation differentially affects mitochondrial function in hCSCs. For this, we treated both hCSCs and MMP9^−/−^ hCSCs with NG and HG for 24 h, and measured their mitochondrial function using the Oroboros advanced bioenergetics platform. We used different drugs that have inhibitory effects on different components of mitochondrial electron transport chain^16^. However, we did not find any significant difference in the mitochondrial function of different complexes in the four groups (Fig. 4f), suggesting that MMP9 may not directly affect mitochondrial function of hCSCs in acute hyperglycemic condition in vitro.

We sought to determine the signaling mechanism underlying hyperglycemia-induced cell death in hCSCs. Mitogen-activated protein kinases (MAPKs) including SAPK/JNK are activated by hyperglycemia^17^. JNK signaling is a hub for programmed cell death^18^. Therefore, we measured the levels of SAPK/JNK in HG-treated hCSCs and MMP9^−/−^ hCSCs. Our results showed upregulated SAPK/JNK in HG-treated hCSCs, which was prevented in MMP9^−/−^ hCSCs (Fig. 4g). To determine the specific role of MMP9 on JNK-induced cell death in hyperglycemic hCSCs, we treated the HG group of both hCSCs and MMP9^−/−^ hCSCs with JNK inhibitor (5 µM final concentration), and measured caspase-3 activity. Our results showed attenuation of HG-induced upregulation of caspase-3 activity by JNK inhibitor in hCSCs (Fig. 4h), supporting the role of JNK signaling in apoptosis of hCSCs. However, the basal level of caspase-3 activity remained unaffected by HG treatment or JNK inhibition in MMP9^−/−^ hCSCs (Fig. 4h), which suggests that MMP9 is upstream to JNK signaling. These findings demonstrates the role of SAPK/JNK signaling in HG-induced apoptosis in hCSCs, and revealed that MMP9 mediates apoptosis in hCSCs.

#### B) Ablation of MMP9 prevents hyperglycemia-induced pyroptosis in hCSCs

**Fig. 5:**
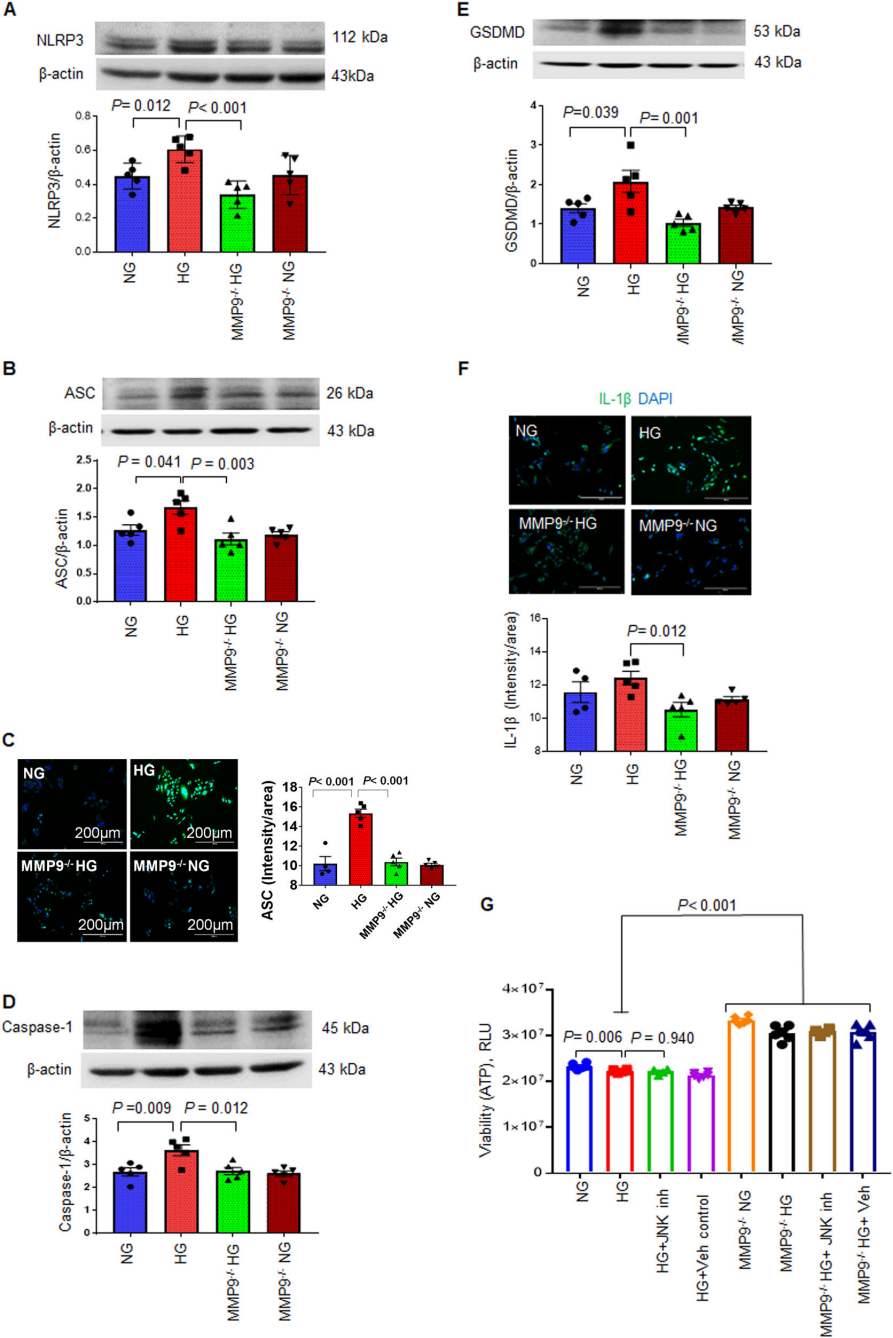
Ablation of MMP9 prevents hyperglycemia-induced pyroptosis in hCSCs. Both hCSCs and MMP9^−/−^ hCSCs were incubated with normal (NG) or hyperglycemic (HG) culture medium for 24 h. These treated cells were used for evaluation of pyroptosis markers. **a**, **b**, **d**, **e**. Western blotting and densitometric analyses of NLRP-3, ASC, caspase-1, and gasdermine D (GSDMD) proteins. β-actin is a loading control. **c**, **f** Immunofluorescence and quantification of intensity of cellular levels of ASC and IL-1β protein. **g** Cell viability evaluation by ATP levels. Increased ATP indicates increased cell viability. Cells were treated with HG or NG in the presence of JNK inhibitor/vehicle. Each dot represents one sample. Values are expressed as mean ± SEM. One-way ANOVA and Tukey’s post-hoc test was used. *P* < 0.05 is considered statistically significant.

Diabetes increases pyroptosis^19,20^, which is initiated by upregulation of NLRP3 (nod-like receptor family pyrin domain containing-3) that forms an inflammasome complex with ASC (apoptosis-associated speck-like protein). ASC activates pro-caspase-1 to caspase-1, the key protein of pyroptosis that activates pro-IL-1β and induces gasdermine D (GSDMD). GSDMD creates pores in the cell membrane resulting in release of inflammatory cytokine IL-1β and pyroptotic cell death^2,4^. To determine the role of MMP9 in hyperglycemia-induced pyroptosis of hCSCs, we incubated hCSCs and MMP9^−/−^ hCSCs with NG or HG medium for 24 h, and measured the key molecular markers of pyroptosis. We found increased levels of NLRP3, ASC, caspase-1, GSDMD, and IL-1β in HG-treated hCSCs (Fig. 5a–f). However, HG-induced upregulation of these pyroptosis markers was prevented by ablation of MMP9 (Fig. 5a–f). These findings demonstrated that a hyperglycemic microenvironment upregulates pyroptosis in hCSCs and that MMP9 mediates hyperglycemia-induced pyroptotic cell death in hCSCs.

#### C) Ablation of MMP9 improves viability of hCSCs irrespective of hyperglycemia

We determined cell viability by measuring the levels of ATP in both hCSCs and MMP9^−/−^ hCSCs treated with either NG, HG, or HG + JNK inhibitor/vehicle. HG treatment decreased ATP levels in hCSCs (Fig. 5g) showing that hyperglycemia decreased hCSCs viability. Decreased viability in the HG group supports increased cell death (Figs. 4a, 5f). However, there was no effect of JNK inhibitor on HG-induced cell viability of hCSCs (Fig. 5g), suggesting a dominant effect of non-apoptotic cell death mechanisms on viability of hCSCs in hyperglycemic microenvironment. Interestingly, cell viability was higher in all treatment groups of MMP9^−/−^ hCSCs as compared to hCSCs (Fig. 5g), demonstrating that suppression of MMP9 improves viability in hCSCs irrespective of a hyperglycemic microenvironment.

### MMP9 mediates cell death by regulating oxidative stress in hCSCs

**Fig. 6:**
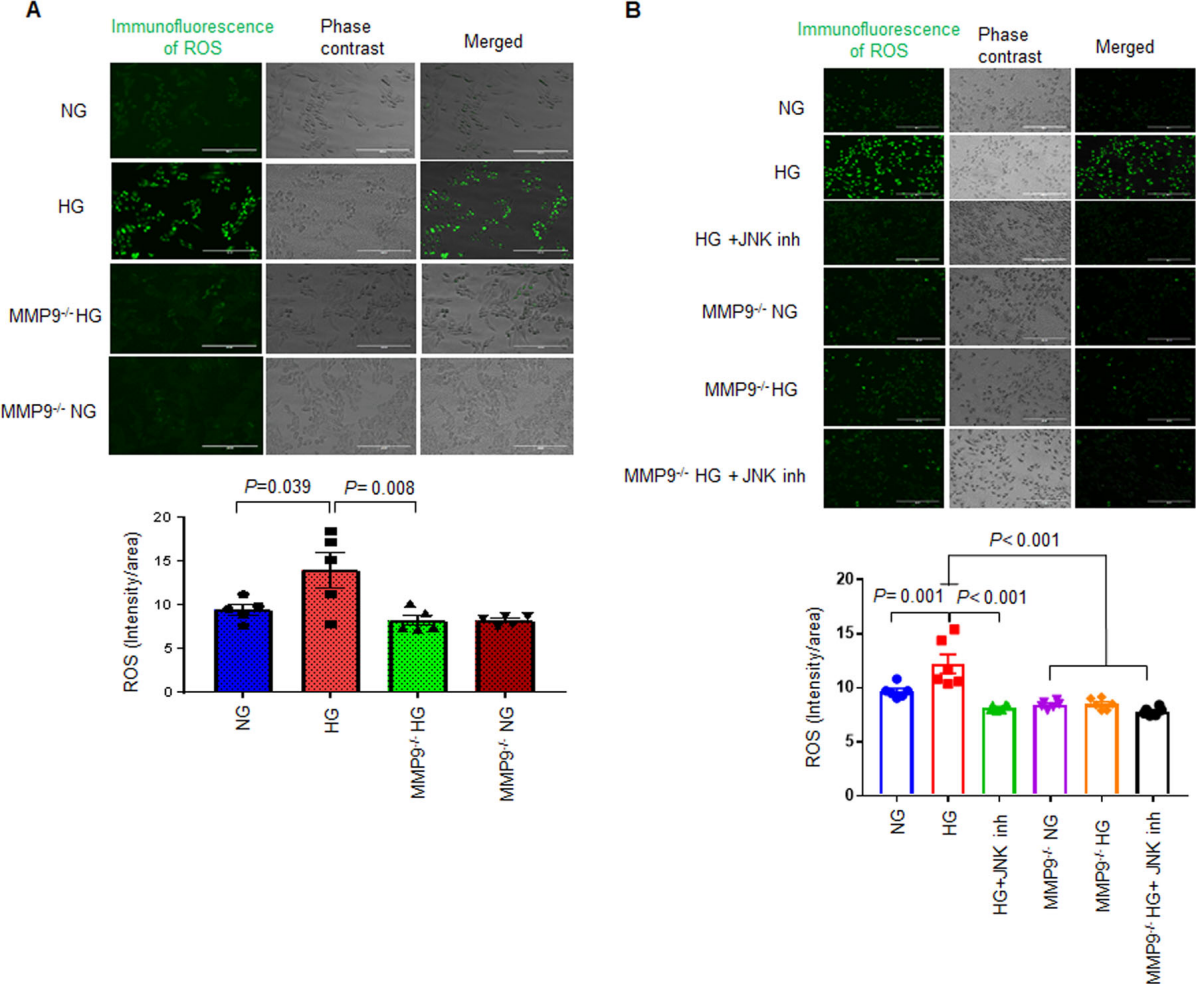
MMP9 mediates hyperglycemia-induced ROS levels in hCSCs via MAPK/JNK signaling pathway. **a** Cellular levels of ROS was measured by CellROX reagent after treating both hCSCs and MMP9^−/−^ hCSCs with normal (NG) or high (HG) glucose. **b** Cellular ROS staining in CSC and CSCMKO cells treated with HG or NG in the presence of JNK inhibitor (5 µM final concentration)/vehicle. Top, representative Immunofluorescence images. Bottom, quantification of cellular levels of ROS (green intensity). Each dot represent single random area from at least three independent wells from a 24-well culture plate. Values are expressed as mean ± SEM. One-way ANOVA and Tukey’s post-hoc test was used. *P* < 0.05 is considered statistically significant.

Hyperglycemia upregulates oxidative stress by increasing the production of reactive oxygen species (ROS)^21,22^. To investigate if MMP9 mediates hyperglycemia-induced ROS production in hCSCs, we treated hCSCs and MMP9^−/−^ hCSCs with NG and HG for 24 h, and measured cellular levels of ROS by CellROX staining. We found increased levels of ROS in HG-treated hCSCs; however, it was prevented in MMP9^−/−^ hCSCs (Fig. 6a). To determine whether ROS generation was associated with MAP/JNK signaling in hyperglycemic condition, we treated the above-mentioned cells with JNK inhibitor and measured ROS levels. ROS production was decreased after JNK inhibitor treatment in the HG group of hCSCs (Fig. 6b), suggesting that the MAP/JNK pathway could be involved in generation of ROS in HG-treated hCSCs. Notably, the expression of ROS remained at basal levels in MMP9^−/−^ hCSCs in all the treatment groups (Fig. 6b), corroborating the fact that MMP9 is upstream to MAP/JNK signaling. These findings revealed that MMP9 plays a pivotal role in mediating hyperglycemia-induced ROS generation in hCSCs.

**Fig. 7:**
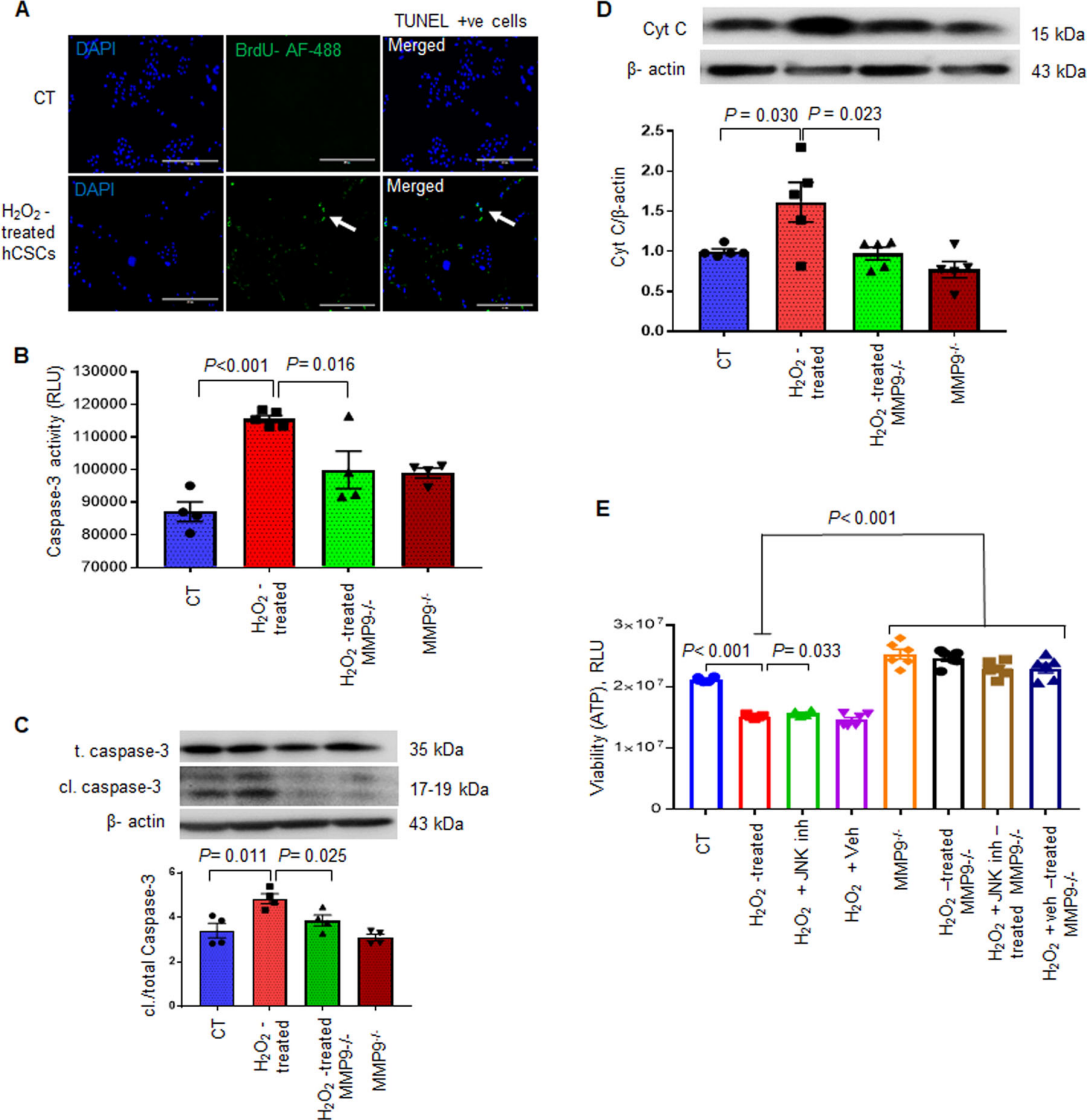
Ablation of MMP9 ameliorates H_2_O_2_-induced activation of apoptosis in hCSCs. Both hCSCs and MMP9^−/−^ hCSCs were incubated with or without hydrogen peroxide (H_2_O_2_) for 24 h. After treatment, they were evaluated for apoptosis. **a** Representative image of TUNEL assay showing increased TUNEL positive cells in H_2_O_2_-treated hCSCs. **b** Caspase- Glo 3/7 assay for caspase-3 activity in H_2_O_2_-treated and control (CT) hCSCs. Relative luminescence unit (RLU) showing caspase-3 activity in different groups. **c** Western blotting and densitometric analyses of cleaved caspase-3 (cl./total caspase-3). **d** Western blotting and densitometric analyses of cytochrome C. **e** Cell viability of cells treated with H_2_O_2_ in the presence of JNK inhibitor/vehicle. β-actin is a loading control. Each dot represents one sample. Values are expressed as mean ± SEM. One-way ANOVA and Tukey’s post-hoc test was used. *P* < 0.05 is considered statistically significant.

ROS-induced oxidative stress promotes apoptotic cell death in stem cells^23^. To determine whether the ablation of MMP9 prevents oxidative stress-induced cell death in hCSCs, we treated hCSCs and MMP9^−/−^ hCSCs with hydrogen peroxide (H_2_O_2_), an inducer of oxidative damage^24^, and evaluated apoptosis and pyroptosis. First, we validated whether H_2_O_2_ increased apoptotic cell death by observing TUNEL positive cells in H_2_O_2_-treated and untreated hCSCs. We found increased numbers of TUNEL positive cells in H_2_O_2_-treated compared to untreated hCSCs (Fig. 7a), demonstrating that H_2_O_2_ increases apoptotic cell death in hCSCs. To examine if this H_2_O_2_-induced apoptotic cell death was mediated by MMP9, we incubated hCSCs and MMP9^−/−^ hCSCs with or without 200 μM H_2_O_2_ culture medium for 24 h. We evaluated apoptosis by measuring expression and activity of caspase-3 and levels of cytochrome c. We found that lack of MMP9 prevented H_2_O_2_-induced upregulation of apoptosis in hCSCs (Fig. 7b, c). The expression of cleaved caspase-3 in MMP9^−/−^ was comparable to control (CT) hCSCs in Fig. 7c, but the activity of caspase-3 by luminiscence method was slightly higher (statistically not significant) in MMP9^−/−^ than CT in Fig. 7b. This could be due to different batches of experimental cells and technical variability in different methods of assessment of caspase-3 activity. Moreover, MMP9 ablation prevented H_2_O_2_-induced cytochrome c upregulation in hCSCs (Fig. 7d), corroborating that MMP9 is required for H_2_O_2_-induced apoptosis in hCSCs. We also measured cell viability by measuring ATP levels in these groups and found that H_2_O_2_-treatment decreased viability of hCSCs, which was prevented by abrogation of the MMP9 gene (Fig. 7e). Altogether, these findings demonstrated a crucial role of MMP9 in mediating oxidative stress-induced apoptosis in hCSCs.

**Fig. 8:**
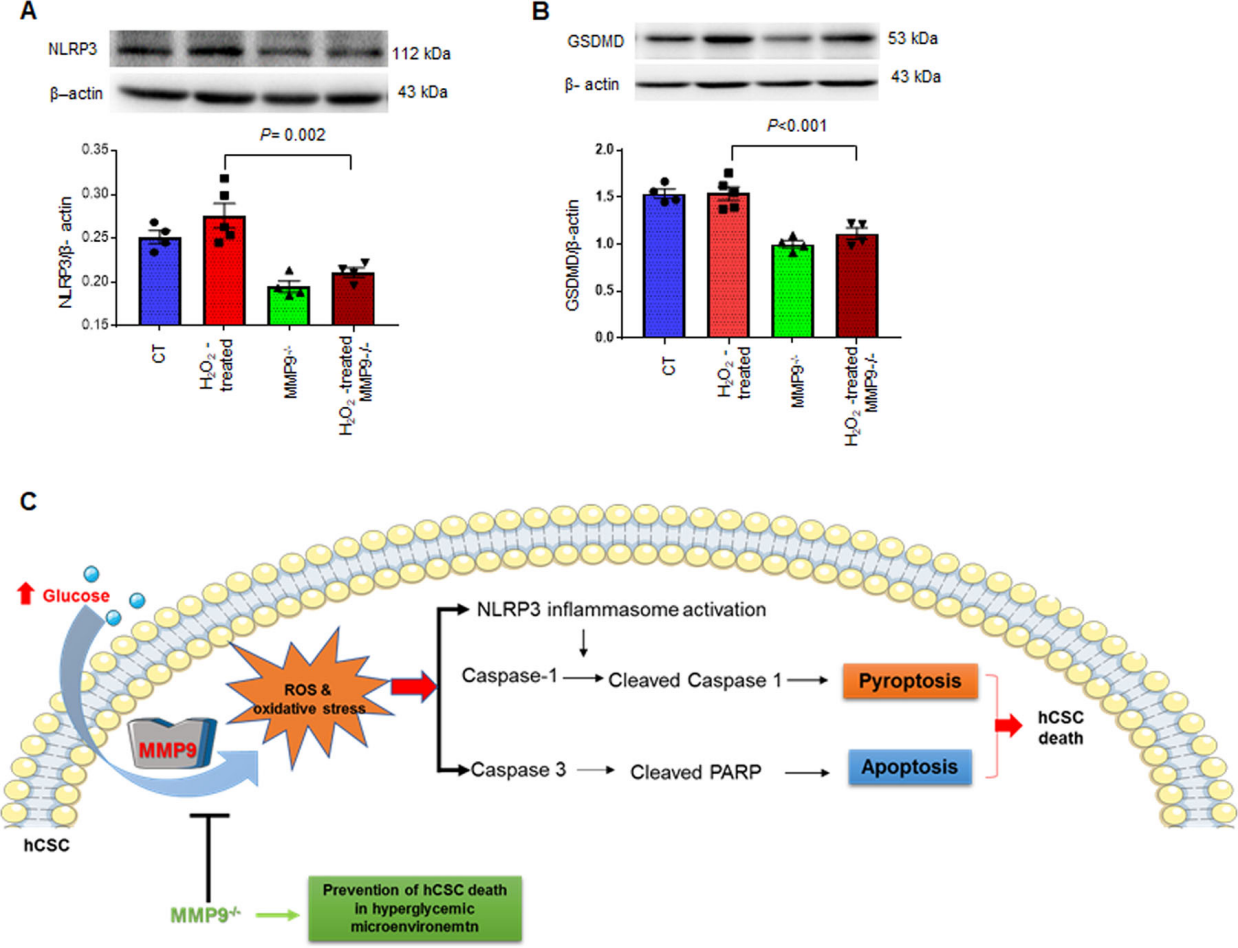
MMP9 is required for H_2_O_2_-induced activation of pyroptosis in hCSCs. Both hCSCs and MMP9^−/−^ hCSCs were incubated with/without hydrogen peroxide (H_2_O_2_) for 24 h, and evaluated for pyroptosis. **a**, **b** Western blotting and densitometric analysis of NLRP3 and gasdermine D (GSDMD) proteins. Values are expressed as mean ± SEM. β-actin is a loading control. Each dot represents one sample. One-way ANOVA and Tukey’s post-hoc test was used. *P* < 0.05 is considered statistically significant. **c** Schematic showing the effects of hyperglycemic microenvironment on ROS production, MMP9 induction and subsequent initiation of apoptosis and pyroptosis in hCSCs, which are prevented by lack of MMP9.

Oxidative stress is a driver of inflammation in the DM heart^25^ and it promotes pyroptotic cell death^26^. Thus, we determined whether MMP9 also mediates oxidative stress-induced pyroptosis in hCSCs. Our results revealed downregulation of pyroptosis markers in H_2_O_2_-treated MMP9^−/−^ hCSCs compared to the H_2_O_2_–treated hCSCs (Fig. 8a–c). Of note, NLRP3 level was drastically (1-fold) decreased in MMP9^−/−^ hCSCs and was unaffected by H_2_O_2_–treatment (Fig. 8a). It indicates that MMP9 is required for initiation of pyroptosis in hCSCs. Similarly, the levels of GSDMD was decreased in untreated MMP9^−/−^ hCSCs compared to hCSCs, and remained unchanged by H_2_O_2_ treatment (Fig. 8b). These findings revealed that MMP9 is indispensable for pyroptosis of hCSCs, irrespective of oxidative stress.

Taken together, we found that hyperglycemia upregulates oxidative stress-induced cell death via apoptosis and pyroptosis in hCSCs, which is mediated by MMP9. Absence/knockdown of MMP9 improves viability of hCSCs by decreasing oxidative stress and suppressing downstream cell death signaling via apoptosis and pyroptosis (Fig. 8d).

## Discussion

DM increases the risk of ischemia and heart failure by ~40%^27^ and decreases long-term survival after acute myocardial infarction (MI)^28^. The efficacy of stem cell therapy is compromised in the diabetic microenvironment^29^. Thus, cardiac repair in the diabetic MI heart is a challenge to current regenerative therapies because the diabetic microenvironment decreases the functional regenerative efficacy of stem cells^29^. Previous studies showed that MMP9 is involved in regulation of stem cell colonization^6^ and differentiation^5^. Moreover, targeted deletion of the MMP9 gene reduces infarct size in ischemia/reperfusion (I/R) injury of the heart^8^. Of note, suppression of MMP9 is associated with reduced apoptosis in the ischemic region of the DM heart in stem cell therapy^30^. Although, detrimental effects of DM on MI and cell therapy have been established, very little is known about how the hyperglycemic microenvironment promotes stem cell death. In the present study, we addressed this issue by measuring hyperglycemia-induced cell death via non-inflammatory apoptotic and inflammatory pyroptotic cell death in hCSCs. In addition, we established that MMP9 mediates cell death via apoptosis and pyroptosis, and ablation of MMP9 improves viability by attenuating cell death in hCSCs.

Several new findings are revealed in the present study. We showed that hyperglycemic microenvironment increases hCSC death via apoptosis and pyroptosis cell death mechanisms. Pyroptosis is triggered by both extracellular and intracellular stimuli^2^. Death-associated molecular patterns, pathogen-associated molecular patterns are extracellular cues that instigate pyroptosis^4^. In our in vitro studies with controlled culture medium, we focused mainly on intracellular cues without external cues to evaluate the specific effects of a hyperglycemic environment on hCSC death. By using several markers, we confirmed that hyperglycemia has an independent effect on promoting cell death via pyroptosis and apoptosis in hCSCs.

We demonstrate that MMP9 is an indispensable mediator of high glucose-induced cell death in hCSCs. Our rationale to target MMP9 was based on previous reports where deletion of one allele of MMP9 reduced infarct size in ischemia/reperfusion injury and deletion of both the alleles of MMP9 further reduced the infarct size^8^, which suggested a potential role of MMP9 ablation in preventing myocardial cell death. Suppression of MMP9 has been associated with improvement of cardiac function in the DM heart, which was transplanted with bone marrow mesenchymal stem cells^30^. Moreover, ablation of MMP9 improves cardiac contractility in the DM heart^12^. Further, the cellular levels of MMP9 increases in the inner hypoxic zone of the embryoid body of ESCs, where the cell death is comparatively higher due to hypoxia. Conversely, MMP9 is downregulated in actively differentiating ESCs where cell survival is high^7^. These findings corroborate the role of MMP9 in stem cell death. However, whether MMP9 acts as a mediator of stem cell death was unclear.

MMP9 may influence stem cell death by extracellular signaling where it increases extracellular matrix stiffness by promoting cardiac fibrosis^31^. Fibrosis decreases the elasticity of extracellular environment and could increase stem cell death^32^. Increased fibrosis also triggers adverse cardiac remodeling following MI, where MMP9 plays a crucial role^33^. Through extracellular signaling MMP9 could increase stem cell death as well as create an adverse myocardial environment that further reduces regenerative capacity of transplanted stem cells. MMP9 is a secretary protein that mostly functions as an extracellular signaling molecule. However, it is also present in the cytoplasm of the stem cell^7^ and in cardiomyocytes^34^. The intracellular regulatory role of MMP9 is largely unknown.

It is reported that MMP9 protein treatment in adult cardiomyocytes isolated from C57BL/6J mice reduces intracellular calcium transient levels and decreases contractility of cardiomyocytes^9^. Cardiomyocytes obtained from MMP9^−/−^ mice have a robust increase in the levels of miR-133a^9^, an anti-hypertrophy^35^ and anti-fibrosis^36^ miRNA, which is decreased in the diabetic heart^37^. Increasing miR-133a in the DM heart improves cardiac contractility^38^ and functions^39^, and prevents DM-induced metabolic remodeling^40^. MiRNA overexpression in the stem cell improves their survival and cardiac regeneration capacity^41,42^. MiR-133a engineered mesenchymal stem cells transplantation augments cell survival and mitigates cardiac dysfunction in the MI heart^10^. Because miR-133a is an anti-apoptotic miRNA^11^, it is plausible that MMP9 could control stem cell death by regulating miR-133a levels.

In the present study, we show that ablation of MMP9 prevents hyperglycemia-induced cell death via apoptosis and pyroptosis in hCSCs and improves viability of hCSCs. We found that MMP9 is the key mediator of caspase-3 activation irrespective of hyperglycemia. The restoration of cleaved PARP and BAD by the ablation of MMP9 gene in hCSCs in both HG and NG-treated cells corroborates that MMP9 is indispensable for apoptosis in hCSCs. Thus, decreasing the levels of MMP9 may be a plausible strategy to mitigate hyperglycemia-induced apoptosis of hCSCs and it could be possibly applicable to other stem cells. Similarly, MMP9 is required for the induction of pyroptosis in hCSCs and ablation of MMP9 blocks pyroptosis in hCSCs irrespective of hyperglycemia. Thus, MMP9 has a pivotal role in hyperglycemia-induced hCSCs death. Altogether, we demonstrate the intracellular role of MMP9 in mediating acute hyperglycemia-induced cell death via apoptosis and pyroptosis in hCSCs.

We reveal that MMP9 mediates ROS-generation in hCSCs. It is known that ROS induces oxidative stress in stem cells^23^, and H_2_O_2_ is an inducer of cellular oxidative stress^24^ that promotes apoptosis^43^ in stem cells. Our results show that hyperglycemia increases ROS production in hCSCs, which is prevented by targeted deletion of the MMP9 gene. Further studies with H_2_O_2_ treatment demonstrate that oxidative stress increases apoptosis and pyroptosis in hCSCs. However, deficiency of MMP9 prevents oxidative stress (H_2_O_2_)-induced apoptosis and pyroptosis in hCSCs. Thus, MMP9 is a key mediator of oxidative stress-induced cell death in hCSCs.

Our selection of model and creation of new model is crucial for delineating the specific role of MMP9 in cell death of hCSCs. We have used hCSCs, which has a more translational value than the rodent-derived CSCs. We have created MMP9^−/−^ hCSCs by the state-of the-art CRISPR-Cas9 technology. Although CRISPR-Ca9 is a straightforward method to knockout genes in vivo, knocking out a gene in vitro using this method is challenging. Our successful creation and validation of MMP9^−/−^ hCSCs was important for determining the specific role of MMP9 in hyperglycemia-induced hCSCs death. Thus, we used a stem cell relevant to humans and created a suitable knockout cell line to examine the specific role of MMP9 as a mediator of cell death in hCSCs.

In conclusion, we reveal that hyperglycemic microenvironment upregulates both non-inflammatory (apoptosis) and inflammatory (pyroptosis) cell death in hCSCs. These two forms of cell death are mediated by MMP9, because ablation of MMP9 prevents high glucose-induced apoptosis and pyroptosis in hCSCs. A key signaling pathway for MMP9-mediated hCSC death could be ROS-induced SAPK/JNK pathway that increases oxidative stress and subsequent cell death. Lack of MMP9 attenuates apoptosis and pyroptosis in normoglycemic hCSCs suggesting that MMP9 is indispensable for cell death in both normoglycemic and hyperglycemic microenvironments. Mitochondria plays a crucial role in both apoptotic and pyroptotic cell death. Our evaluation of mitochondrial function demonstrated that acute hyperglycemia or MMP9 ablation does not have significant effect on mitochondrial function in hCSCs.

Although our findings provide a novel intracellular role of MMP9 in mediating cell death via apoptosis and pyroptosis in hCSCs, further studies on other types of stem cells are needed to establish that MMP9 inhibition decreases cell death and improves viability of stem cells in hyperglycemic microenvironment. Future in vivo studies using a diabetic MI model with/without MMP9^−/−^ will establish whether MMP9 suppression could be a potential therapeutic strategy to improve stem cell viability in regenerative therapy of the diabetic MI heart.

## Materials and methods

### Human cardiac **s**tem cells (hCSCs) and culture medium

We have commercially procured hCSCs from Creative Bioarray Company (CSC-0030). As per the company website (https://www.creative-bioarray.com/QualiCell®-Human-Adult-Ventricular-Cardiac-Stem-Cells-XLC452-CSC-C00305-item-2861.htm), the source of these cells are human adult ventricular cardiac tissue and they are positive for Sca-1^+^, c-kit, Nkx2.5, MEF2C (myocyte enhancer factor-2c) and other markers. We cultured these cells in commercially available medium (CM 1330Z) from the same company. In addition, we have used Phosphate Buffered Saline (Lonza), 0.1% Trypsin-EDTA (Sigma) for cell culture.

### Generation of MMP9^−/−^ hCSCs by CRISPR-Cas9

We seeded 1 × 10^6^ cells in a 6-well plate in complete medium. After 24 h, cells were co-transfected with 1 μg of MMP9 specific CRISPR-Cas9 (GFP-tagged) and 1 μg of HDR (RFP-tagged) plasmid along with Lipofectamine 2000 in OptiMEM and incubated at 37 °C in a CO_2_ incubator overnight. The medium was replaced with complete medium and cells were incubated for another day. The cells were observed under a fluorescence EVOS microscope for transfection efficacy and expression of RFP, GFP, and both expressing cells. These cells were used for fluorescence-activated cell sorting (FACS) to obtain the double positive (GFP and RFP) MMP9^−/−^ hCSCs (Fig. 3).

### Treatments groups

We treated cells with either high glucose (HG), normal glucose (NG), or hydrogen peroxide (H_2_O_2_). We incubated cells with 25 mM of d-glucose (HG) to create a hyperglycemic microenvironment. As a control, we used 5 mM of d-glucose (NG), which is comparable to physiological levels of glucose. To balance osmolality, we added 20 mM of mannitol to the 5 mM glucose. For H_2_O_2_ treatment, we incubated cells with 200 µM H_2_O_2_. The total duration of treatment was 24 h.

### Immunocytochemistry

We used a 24-well culture plate to seed 10,000 cells. After 24 h, we fixed them with 10% formalin for 20 min and then washed them with ice-cold phosphate buffered saline (PBS) three times with 5 min intervals. For permeabilization, we kept these cells in 0.1% triton X-100 for 10 min and then washed them with ice-cold PBS three times with 5 min intervals. Cells were then incubated with primary antibodies overnight at 4 °C. The primary antibodies used were Abcam antibody MEF2C (1:100, ab191092), IL-1β (1:100, ab9722); Santa Cruz antibody Nkx2.5 (1:50, sc-8697), β-MHC (1:50, sc-53089), ASC (1:50, sc-54414); and Millipore antibody Sca-1 (1:50, AB4336). After washing the cells with ice-cold PBS three times with 5 min intervals, they were incubated with fluorescence-tagged secondary antibody for 1 h at RT. The secondary antibody dilution was two times of primary antibody dilution. The Invitrogen secondary antibodies used were anti-rabbit Alexa Fluor 594 (A11012), anti-rabbit Alexa Fluor 488 (A21441), anti-mouse Alexa Fluor 594 (A21201), and anti-mouse Alexa Fluor 488 (A 21200). After washing with ice-cold PBS three times with 5 min intervals, they were counterstained with 1 µg/ml DAPI for 3–5 min, washed one time with PBS, and observed under an EVOS microscope.

For quantification of intensity of fluorescence, we used NIH Image J software. We followed the previously established protocol for the basic intensity quantification by normalizing mean gray intensity against cell area^40^. The full analyses protocol is available at the following link: https://www.unige.ch/medecine/bioimaging/files/1914/1208/6000/Quantification.pdf.

### Protein isolation and western blotting

To extract protein, we incubated cells with radio-immuno-precipitation assay lysis buffer (BP-115D, Boston BioProducts) for 30 min on ice. The cell lysate was centrifuged at 10,000 rpm at 4 °C for 20 min and supernatant was collected for protein estimation using BCA method (Pierce BCA Protein Assay Kit, 23227, Thermo Fisher). Thirty microgram of protein was loaded for SDS PAGE. Positively charged nitrocellulose membrane was used for protein transfer. Membrane was blocked with 5% nonfat milk for 1 h and incubated with primary antibody overnight at 4 °C on a rocker. The primary antibodies used were Abcam antibody MEF2C (1:1000, ab191092), GSDMD (1:1000, ab209845), BAD (1:1000, ab32445) and β-actin (1:5000, ab56164); Santa Cruz antibody Nkx2.5 (1:500, sc-8697), MMP9 (1:500, sc-393859) and ASC (1:500, sc-54414); Cell Signaling Technology antibody caspase-3 (1:1000, 9662S), cleaved caspase-3 (1:1000, 9664), PARP (1:1000, 9532S), cleaved PARP (1:1000, 94885S), SAPK/JNK (1:1000, 9258s), NLRP3 (1:1000, 15101 for Fig. 8a); and Novus antibody NLRP3 (1:1000, NBP2-12446, for Fig. 5a), caspase-1 (1:500, NBP1-45433). The membrane was washed with TBST three times with 5 min intervals. They were incubated with secondary antibody for 1 h at RT. The dilution of the secondary antibody was double of the primary antibody dilution. The secondary antibodies used were Cell Signaling Technology, anti-rabbit IgG-HRP (7074S) and anti-mouse IgG-HRP (7076). Blots were developed using Clarity™ Western ECL Substrate (Bio-Rad Laboratories, 1705061) and Chemidoc (Bio-Rad Laboratories) instrument, and band intensity was analyzed by ChemiDoc software (Bio-Rad Laboratories). Membranes were stripped with Restore™ PLUS Western Blot stripping buffer (Thermo Scientific Inc., 46430).

### TUNEL assay

We used APO-BrdU TUNEL Assay kit (Thermo Scientific, A23210) and followed the kit protocol. In a 24-well plate, 1 × 10^4^ cells were incubated with NG or HG for 24 h. The positive control cells were treated with 30 ng/ml TNF-α and 10 µg/ml of cyclohexamide for 12 h. Cells were then fixed with 1% paraformaldehyde (dissolved in PBS) on ice for 15 min and washed with ice-cold PBS three times with 5 min intervals. We dehydrated the cells with ice-cold 70% ethanol (v/v) for 30 min on ice or in a −20 °C freezer. We prepared 50 μl per sample DNA labeling solution by mixing adequately 10 μl of reaction buffer, 0.75 μl of TdT enzyme, 8.0 μl of BrdUTP, and 31.25 μl of dH_2_O. Cells were incubated with 50 µl of DNA labeling solution for 60 min at 37 °C. They were washed by adding 300 μl of rinse buffer three times with 5 min intervals. Another set of 100 μl antibody staining solution for each sample was prepared by mixing 5 μl of Alexa Fluor 488 dye-labeled anti-BrdU antibody with 95 µl of rinse buffer. Cells were incubated with this 100 μl of fluorescence labeled antibody solution for 45 min at RT in dark and then washed. They were counterstained with propidium iodide/RNase A staining solution for 20 min at RT or DAPI staining for 5 min. After one-time washing with PBS, cells were kept in 200 μl PBS. They were observed under an EVOS microscope and imaged.

### Flow cytometry for Annexin V and DAPI staining

We determined apoptosis by Annexin V and DAPI staining using FITC Annexin V apoptosis detection kit (BD, 556547). We have used DAPI instead of PI for nuclear staining because our MMP9KO cells are having red florescence transfected plasmid (RFP) which was causing interference with PI staining. We incubated 0.4 × 10^6^ hCSCs and MMP9^−/−^ hCSCs in a 6-well plate with HG and NG medium as explained above for 24 h. After treatment, mild trypsin/5–10 mM EDTA solution was applied to the cells to detach them. These cells were then washed with ice-cold PBS twice and re-suspended in 1× binding buffer. The number of cells were scored using a cell counter (BioRad) and 1 × 10^5^ cells were transferred to a 5 ml polystyrene tube with 100 µl of binding buffer, 5 µl of FITV Annexin V, and 5 µl of 4′,6-diamidino-2-phenylindole (DAPI). These solutions were mixed by either pipetting or mild vortex with the cells and the tubes were incubated for 20 min at RT in dark. At the end of incubation, 400 µl of 1× binding buffer was added to the tube and these cells were used for flow cytometry. We used flow cytometry core facility at the University of Nebraska Medical Center and analyzed the results by Flow Jo software.

### In-gel gelatin zymography

We used 10 µg of cell medium for in-gel gelatin zymography. A 10% SDS-polyacrylamide gel containing 0.1% gelatin and another standard 10% SDS polyacrylamide gel without gelatin were used for electrophoresis of the protein. The gel without gelatin was stained with coomassie and imaged to assess equal protein loading. The gelatin containing gel was developed for MMP9 activity. The gelatin-gel was washed with water for 5 min and incubated in renaturation buffer [2.5% (v/v) Triton X-100 in MQ water] for 90 min at RT. The renaturation buffer was replaced with a fresh renaturation buffer at the interval of 30 min. The gel was then incubated in activation buffer (50 mM Tris-HCl, pH 7.5, 5 mM CaCl_2_, 0.2 M NaCl, and 0.02% Brij-35) for 48 h at 37 °C on a slow rocker. It was washed one time with water for 5 min and then stained with 0.2% Coomassie Brilliant Blue R-250 for 10–15 min, until complete staining of the gel. The gel was washed with distilled water, and imaged by Chemidoc (BioRad).

### Caspase-Glo 3/7 assay

To determine caspase-3 activity in the cells, we used caspase-Glo 3/7 assay kit (Promega, G8090) and followed the kit instructions. Briefly, 0.4 × 10^6^ cells seeded in a 6-well plate were treated with NG or HG in incomplete medium for 24 h in a cell culture incubator at 37 °C with 5% CO_2_. The cell lysates were collected using RIPA buffer and 100 μl of cell lysate was transferred in each well of a 96-well plate, which is white-walled multi-well luminometer-compatible. Equal volume of caspase-Glo 3/7 reagent was added to the cell lysate in the well and the plate was covered and sealed. The plate containing the cell lysate and the reagent was kept on a plate shaker at 300 rpm for 30 s, and then incubated for 1 h at RT in dark. The luminescence of each well was measured using luminometer (Promega, GLOMAX Multi Detection System). Cell culture medium was used to measure background reading. Background reading was subtracted from the end-point reading of each sample. Values were presented as relative luminescence intensity (RLU).

### Pretreatment of cells with JNK inhibitor and measurement of caspase3/7 activity

To determine the activation of caspase3/7 under HG condition and its inhibition by JNK inhibitor, 5 × 10^3^ cells were seeded in 96-well plate in complete medium in cell culture incubator at 37 °C with 5% CO_2_. After 24 h of incubation, cells were treated with HG or NG as well as HG with JNK inhibitor or vehicle control. We pretreated the cells with 5 µM of final concentration of JNK inhibitor for 30 min before treating them with HG or NG. Cells were treated with HG or NG for 24 h. At the end of treatment, cell death was measured using Caspase-Glo 3/7 assay kit (Promega, Cat #G811A) by measuring luminescence as per assay protocol. Medium without cells were used as a background luminescence. Values were presented as a RLU.

### Pretreatment of cells with JNK inhibitor and measurement of viability

To measure the viability of CSC and CSCMKO cells treated with HG or NG in the presence or absence of JNK inhibitor, 5 × 10^3^ cells were seeded in 96-well plate in complete medium in cell culture incubator at 37 °C with 5% CO_2_. After 24 h of incubation, cells were treated with HG or NG as well as HG with JNK inhibitor or vehicle control. We also measured the viability of cells treated with/without H_2_O_2_. We pretreated the cells with 5 µM of final concentration of JNK inhibitor for 30 min before treating them with HG, NG, or H_2_O_2_. Cells were treated with HG, NG, or H_2_O_2_ for 24 h. At the end of treatment, cell viability was determined using CellTiter-Glo Luminescence Cell viability Assay kit (Promega, Cat #G7571) by measuring luminescence (ATP level) as per assay protocol. Medium without cells were used as background luminescence. Values were presented as a RLU.

### ROS measurement

To measure ROS generation in the HG or NG treated cells, we added 5 µM CellROX reagent (Life technology, C10444) at the final concentration of culture medium, and incubated for 30 min at 37 °C in CO_2_ incubator. After incubation, cells were fixed with 3.7% formaldehyde for 15 min, washed with PBS three times. Cells were observed and imaged under an EVOS microscope with 488/522 excitation/emission.

### Mitochondrial respiratory complex functional assessment

Cell mitochondrial respiration was assessed using Digitonin-permeabilized cell methods in mitochondrial respiration medium (MiR05) containing 0.5 mM EGTA, 3 mM MgCl_2_·6H2 O, 20 mM taurine, 10 mM KH2-P04, 20 mM HEPES, 1 g/L BSA, 60 mM potassium-lactobionate, 110 mM sucrose, pH 7.1, while being continuously stirred, at 37 °C using a high-resolution Oxygraph-2k (Oroboros, Austria, Innsbruck) as previously described^16^. Experiments evaluating intact cell respiration were performed with hCSCs or MMP9^−/−^ hCSCs suspended in culture medium consisting of low glucose Dulbecco’ s Modified Eagle Medium (DMEM; GIBCO) containing 1000 mg/L glucose and 110 mg/L sodium pyruvate. Without substrate addition, routine respiration was determined. All respiratory states were corrected for residual/non-mitochondrial oxygen consumption (0.5 μM rotenone and 2.5 μM antimycin A). Routine respiration was identified in MiR05 mitochondrial medium. State 2 respiration was assessed by the addition of complex I substrates glutamate (10 mM) and malate (2 mM). ADP (5 mM) induced the state 3 respiration/maximal oxygen consumption. A subsequent titration of succinate (10 mM) allowed for evaluation of complex I + II state 3 respiration. Cytochrome c was used for mitochondrial quality control. Carbonyl cyanide-4-(trifluoromethoxy) phenylhydrazone (FCCP, 0.6–1 μM) and potassium cyanide (KCN, 10 mM) were used for Complex IV respiration. The samples were blinded for analyses of mitochondrial functions.

### Statistical analysis

All the experiments were replicated three times using different culture plates and repeated at least twice. At least 5 culture wells were used for one experiment. The values are expressed as mean ± SEM. To compare the difference of mean among the four experimental groups, we used one-way analysis of variance (ANOVA), which was followed by Tukey’s post-hoc test for multiple comparison between the groups. To compare the mean of only two groups, we used student *t*-test. The *P* value is obtained from two-tailed analyses. All the statistical analyses were performed using Graph Pad Prism7 software. A value of *P* < 0.05 is considered statistically significant.
